# Multitrophic dataset for multi-taxon abundance and richness estimations from biomass and nutrient energetics

**DOI:** 10.1038/s41597-026-07997-4

**Published:** 2026-08-05

**Authors:** Elizabeth Baach, Carsten F. Dormann

**Affiliations:** Biometry & Environmental System Analysis, University of Freiburg, Freiburg im Breisgau, Germany

## Abstract

Ecosystem dynamics are driven by the interactions of the species within. Trophic interactions are structured by organisms’ acquisition and conversion of resources into biomass. This shapes species abundance and richness through variation in resource availability, competition, and trophic cascades. Despite the connections between trophic webs and biodiversity, data within and between these fields remain taxonomically siloed. We compile and organize data from ecological energetics, species-traits, nutrient allocation, and biodiversity research into unified, navigable datasets that bridge these research domains. These datasets focus primarily on temperate forest systems, although data from other ecosystems are additionally included. They include species-level variables relating to tree biomass and nutrient (nitrogen and phosphorus) allocation, alongside species-level consumption (attack rate), assimilation (assimilation efficiency and nitrogen assimilation efficiency), and body mass and nutrient content data for birds, invertebrates, and mammals across multiple trophic groups. Combined with group-level abundance-richness relationships, these data enable biomass- and nutrient-based trophic web development and estimation of species abundance and richness.

## Background & Summary

Organisms interact in various manners, with one of the most basic and universal being through consumption. These consumption events can be aggregated and organized into food webs. Trophic webs vary in complexity and level of detail based on the system for which they are constructed and the aim behind its development^1^. One way trophic webs can be built is through the use of ecological energetics that consider the energy costs associated with organismal activities in their respective environments^2,3^. These costs vary between trophic and taxonomic groups, depending on the quality of consumed resources as well as the traits of the organisms themselves^4^.

The quantification of trophic webs can elucidate numerous ecosystem properties, particularly those related to population dynamics. There are different forces (e.g., bottom-up, top-down) through which trophic interactions can determine population structure^3,5^. Understanding these connections, and the further relationship to species richness is promoted through access to data that researchers can utilize to quantify them. Biomass allows for straight-forward quantification of trophic interactions, as this can be considered the currency exchanged through these interactions. However, consideration of nutrient limitation in place of biomass limitation may allow for more precise evaluations of food webs due to nutrient imbalances between trophic levels^4^. Availability of biomass and nutrient data is limited and scattered across disparate fields, thus their compilation facilitates researchers to find and apply these values to explore different aspects of trophic interactions.

Biomass and abundance values can be used to estimate species diversity, most often as species richness, through species abundance distributions and rarefaction curves^6,7^. These methods can be influenced by the body size, or mass, of the considered organisms as well as the sampling area, both of which are included in our data. The relative rarity of different species can play an important role in these relationships^8^, and we suggest users of this data to consider this when performing analyses as this is not implicitly included in the data.

Here we present datasets comprised of variables that can be used to make biomass- or nutrient-based multitrophic budgets and paired to species abundance and richness estimations. These data span multiple disciplines from organismal energetics and physiology to biodiversity monitoring. The compilation of these data was motivated through the desire to connect resource availability to species abundance and richness, based on the species-energy theory^9,10^ and the more individuals hypothesis^11^. Despite the mechanistic connections between ecological energetics and species abundance and richness, these data are previously not well linked and come from widely diverse sources. Thus, our aim is to provide a comprehensive collection of data that can be used for further exploration of trophic and biodiversity dynamics, particularly in temperate forests.

## Methods

This is a compilation of empirically collected data. It includes data that, to our knowledge, were not previously digitized. These data were originally collected with a focus on temperate forest systems; however, we include data outside of this system in the datasets. All references used are cited alongside each data value with links (mainly as a doi) available for each listed citation in the references tab of each file.

We used Google Scholar to search the literature for each of the considered values directly. We additionally searched for works available in the University of Freiburg library system. While some sources report data relevant to multiple datasets, our search was generally for the specific values. Our intention for this data collection was to synthesize previously disparate data that are ecologically tied, as well as make these data more accessible through digitization and compilation. Data were collected and compiled manually from the source and converted when relevant. We describe specific search and processing for each dataset below.

For the *Primary Producers* data, we searched for literature on tree biomass and nutrient allocation. These data are generally found in the forestry literature and have been used to fit tree allometric functions. We used the general search terms such as “tree biomass allocation” or “tree nitrogen allocation” to find suitable data. Because data covering tree fruit were sparse we performed an additional search specifically for allocation to fruit. As our initial aim had a focus on temperate forests, we looked for sources specific to this ecosystem. In this way we excluded biomass and nutrient allocation data for trees outside of the temperate (and temperate-boreal) zone (e.g., tropical rainforests or xeric shrublands) and made note of special forest conditions (e.g., management treatments or regeneration status) in the notes column. We included data that reported biomass, nitrogen, or phosphorus allocation to different tree parts, excluding reports of other nutrients (e.g., potassium, sodium) that were additionally reported in some cases. We report all biomass values in tonnes/hectare and thus converted those not originally reported with this unit, identifying the conversion in the notes column. Some reports include fruit reported as 0; however, when the source did not indicate if that meant there was no fruit on the tree or that they chose to exclude fruit measurement from reports, we marked this component with “-“ and indicate this change in the notes column. We report multiple units in the nutrient allocation dataset; however, we standardized all values to follow metric units of mass (mg, g and kg) and converted all area measurements to be at the hectare scale to match the biomass allocation dataset. Many original reports did not need conversion, and for the few that did we make note of the original units in the notes column.

To compile the *Consumers* data, we searched specifically for the values of interest, using search terms such as “assimilation efficiency” and “attack rate”, in general as well as for the taxonomic and trophic groups we include (birds, mammals, invertebrates). We additionally searched for literature using common terms similar to our target variables that we found in the literature, for example “production efficiency”, “digestion efficiency” and “consumption rate”. We carried out the same process for “nitrogen assimilation efficiency”, where literature additionally reporting “nitrogen use efficiency” and “nitrogen digestibility” were also evaluated. To find the nutrient composition of consumers, we searched for the taxonomic groups we consider using “nutrient composition”, “nitrogen composition” and “phosphorus composition”. We also searched through the ecological stoichiometry literature and the citations common within this field. For all of the above datasets, when we found suitable data the related works generally did as well, we therefore evaluated other works by the same authors, the works that were cited, and the works that cited the source (i.e., citation chaining and backward citation searching). We excluded and filtered out taxonomic groups that we do not consider (e.g., amphibians, reptiles) as well as non-terrestrial groups. However, some included species (particularly birds) are associated with aquatic environments and we make note of that in the data. We standardized assimilation efficiency (AE), nitrogen assimilation efficiency (NAE), and all but 1 row of the nutrient content values to be reported as a percent, and noted the original units in the cases where conversions were made. In the one case, we were unable to find enough information about the measurements to convert them. We standardized attack rate values to be as a percent or in metric mass/time (e.g., g/day, kg/year), to best fit the biomass allocation dataset. We note any conversions made to the original values, with 5 measurements reported using kcal instead of a metric mass as the caloric values of the food items were unavailable.

The *Abundance richness* data were compiled with a focus on the taxonomic and trophic groups we include as well as on temperate forests. For the individual mass data, we first consulted the sources used for attack rate, assimilation efficiency, and nutrient content. For groups that did not have individual mass data from sources used in the other datasets, we searched for mammals and birds using species names from EltonTraits^12^ as they make note of the diet classification of organisms, which we use a grouping characteristic (trophic level and trophic niche). For invertebrates we searched using “individual mass” or “individual weight” with “invertebrate”, “insect” and “arthropod” to find sources reporting these data. The process was similar for the abundance richness dataset: beginning first with already included sources that report abundance and richness. We then searched for a database that would include abundance and richness data for temperate forests, leading to the consideration of data from the PREDICTS database^13^. We additionally searched for literature using “species abundance relationships”, “species abundance curves (SACs)”, and “abundance richness relationships” both in general as well as with the names of the considered taxonomic groups (birds, mammals, invertebrates). We excluded values for taxonomic groups we do not consider, and cases where the taxonomic group was not reported or classified too broadly (e.g., as Animalia). For the abundance richness data, there were cases of abundances less than 1 or less than the reported richness value, which we excluded in addition to all missing or NA values. We also excluded data from sources that did not report area with abundance and richness values, making an exception for^14^ that fit species-abundance curves (SACs) as their express goal behind data collection was to fit abundance to richness (n = 4 rows), with this being mentioned in the notes column. Extra processing was necessary for data sourced from the PREDICTS database^13^. These data are available directly; however data processing tools are available to assist with filtering the original, very large, dataset^15^. We recommend these tools to whomever is unfamiliar with PREDICTS and wishes to source data directly in order to better understand data structure and facilitate processing and use. To fit the original aims of this dataset, we followed steps outlined by^15^ to filter the data to specific biomes that fit into the general classification of temperate forest. As was done for all data in this dataset, we excluded values pertaining to taxonomic groups outside of our consideration and that did not have acceptable abundance values, as described before.

## Data Records

There are three main sets of compiled data, each containing multiple datasets within (Table 1). This data compilation is hosted in Zenodo^16^ [10.5281/zenodo.18630029] where we offer users multiple formats to access the data: three Excel (.xlsx) sheets that each contain several datasets within, these same files are additionally provided as an OpenDocument Spreadsheet (.ods). We also offer these data as Comma-Separated Values files (.csv) grouped using zipped folders, as Metadata and References are grouped by data sheet. We define column names and values in the Metadata tab and include more detailed links to citations in the References tab of each spreadsheet.

**Table 1:** Summary of data presented including the number of observations and the number of species represented by these observations.

Dataset		Number of observations	Number of species
Primary Producers	biomass allocation	3024	87
	nutrient allocation	952	56
Consumers	attack rate	527	137
	assimilation efficiency	1378	309
	nutrient content	563	406
	NAE	364	53
Abundance richness	individual mass	1092	648
	abundance richness	2170	*37

*In the case of the abundance richness dataset the number of groups represented is reported (see text for details).

The *Primary Producers* spreadsheet includes two main datasets: biomass allocation and nutrient allocation. Biomass allocation includes the dry biomass (tonnes/hectare) of different measured tree parts, specifically stem wood, stem bark, branches, fruit, foliage, and roots. We specify the species and tree identity for each tree part measured to distinguish between species and individuals. This dataset also includes descriptions of the forest where the tree measurements were conducted, including location (country or region), forest species composition, age of the forest, tree density (trees/hectare), volume of trees (basal area in square meters/hectare), and coverage of leaves (Leaf Area Index). The nutrient allocation dataset includes the distribution of nitrogen (N) and phosphorus (P) to different tree parts and includes the same identification factors used in the biomass allocation dataset: individual tree ID, tree species name, and location. This dataset includes values for biomass of tree parts and the units for which that is measured and the concentration of N and P for a given tree part and their respective units.

The *Consumers* spreadsheet includes, in addition to the Metadata and References, four datasets: assimilation efficiency, attack rate, nutrient content, and nitrogen assimilation efficiency (NAE). All of these datasets include four standard columns for identifying and classifying organisms: taxonomic category (bird, mammal, invertebrate), name (scientific name or smallest possible taxonomic classification), family (taxonomic family), trophic level (herbivore, omnivore, carnivore), and trophic niche (diet-based category of the reported food consumed by the organism, e.g., folivore, granivore, insectivore). The assimilation efficiency dataset reports assimilation efficiency (AE) as a percent, representing the amount of consumed biomass incorporated into the biomass of the organism. There is variation in how AE is estimated and reported (e.g., digestive efficiency, absorption efficiency, metabolizable energy coefficient) and we note such differences in the notes column. Some of these values, including the Efficiency of Conversion of Ingested food (ECI) and productive efficiency, account for organismal growth and losses to respiration and users wanting to account for biomass assimilation should be aware of this. See also the notes column of this dataset for more specific details on the calculation of these values, when not directly reported as a percent. The attack rate dataset includes a column for the attack rate value as well as one to indicate in which units the attack rates are reported (e.g., %, g/day, kcal/day). There is also a column that reports, when available, the diet of the given organism as reported by the study. The nutrient content dataset reports the content of nitrogen (N) and phosphorus (P) of a given organism’s body, as well as the units in which these values are reported (e.g., C:N, C:P, %). The nitrogen assimilation efficiency (NAE) dataset reports values for the relative amount of nitrogen, or protein, incorporated into the body compared to nitrogen or protein intake. These values are all reported as a percent, and indicated as a measure of nitrogen or protein in another column. See also the notes column of this dataset for more specific details on the calculation of these values, when not directly reported as a percent.

The *Abundance richness* spreadsheet includes two datasets in addition to the Metadata and References sheets: individual mass and the abundance richness data (abun rich). Both of these datasets include the common columns included in the datasets of the Consumers spreadsheet: taxonomic category (bird, mammal, invertebrate), trophic level (herbivore, omnivore, carnivore), and trophic niche (diet-based category of the reported food consumed by the organism, e.g., folivore, granivore, insectivore). The individual mass dataset reports the mass of an individual of a given organism in grams as well as the name (scientific name or smallest possible taxonomic classification) and the family (taxonomic family). The abundance richness dataset reports group as the general taxonomic group to which the organisms belong, rather than name and family, as these data relate to many species. This dataset reports the number of individuals and the number of species represented by these individuals as well as the area for which these values are reported and the units of this area value. Habitat is additionally included in the abundance richness dataset and identifies the general ecosystem type, as reported by the sources.

## Technical Validation

There are no additional steps users need to take to validate these data as they are a compilation of empirically collected values. Should users of these data need more details about the specifics for any given value, we direct them first to the notes column of the dataset as well as the reference(s) associated with the data.

## Usage Notes

We recommend using the common columns (e.g., trophic level, taxonomic category) to connect values across datasets. The name and family column may contain outdated values, based on what was reported in the literature, so users should be aware of this when connecting data at the species- or family-level from various sources. We did our best to update scientific names and taxonomic families based on current classifications and encourage users to make note of this.

For example, using R^17^, users can join data by the values of these columns using the “mutating join” functions (e.g., left_join, full_join) in the dplyr package^18^. This allows users to merge data with matching values in these columns to connect values across datasets.

## Data Availability

These data are available on Zenodo (10.5281/zenodo.18630029) where users find the three main spreadsheets (.xlsx and.ods) containing the datasets described above. The data are additionally available individually as CSV files grouped in their respective folders.

## Code availability

No code was developed to generate these datasets. For code useful in processing data from the PREDICTS database, see^13,15^.
